# Roll‐Coated Fabrication of Fullerene‐Free Organic Solar Cells with Improved Stability

**DOI:** 10.1002/advs.201500096

**Published:** 2015-04-28

**Authors:** Pei Cheng, Huitao Bai, Natalia K. Zawacka, Thomas R. Andersen, Wenqing Liu, Eva Bundgaard, Mikkel Jørgensen, Hongzheng Chen, Frederik C. Krebs, Xiaowei Zhan

**Affiliations:** ^1^ Beijing National Laboratory for Molecular SciencesCAS Key Laboratory of Organic SolidsInstitute of ChemistryChinese Academy of SciencesBeijing100190P. R. China; ^2^ Department of Energy Conversion and StorageTechnical University of DenmarkRoskildeDK‐4000Denmark; ^3^ University of Chinese Academy of SciencesBeijing100049P. R. China; ^4^ Department of Polymer Science and EngineeringZhejiang UniversityHangzhou310027P. R. China; ^5^ Department of Materials Science and EngineeringCollege of EngineeringKey Laboratory of Polymer Chemistry and Physics of Ministry of EducationPeking UniversityBeijing100871P. R. China

**Keywords:** fullerene‐free, nonfullerene acceptor, organic solar cells, roll‐to‐roll, stability

## Abstract

**Large area, fullerene‐free organic solar cells** with improved stability and efficiency of up to 1% are fabricated by the roll‐coating process on indium tin oxide free and flexible substrates, under ambient conditions.

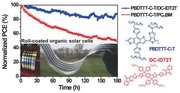

The solar cell is one of the best candidates for clean energy and sustainable development. Especially, the bulk heterojunction (BHJ) organic solar cells (OSCs) are promising because of some advantages, such as simple preparation, low cost, light weight, and large area flexible fabrication. This has been the focus of research in the past decade.^1^, ^2^, ^3^, ^4^, ^5^, ^6^

Nowadays, fullerene derivatives (typically PC_61_BM and PC_71_BM) are widely used as electron acceptors in OSCs because of ultrafast photoinduced charge transfer at donor/acceptor (D/A) interfaces,^7^ high electron mobility,^8^ and good ability to form favorable nanoscale networks with donor materials.^9^ BHJ OSCs based on interpenetrating networks of semiconducting polymers and fullerene derivatives exhibited the best performance with power conversion efficiencies (PCEs) exceeding 11%.^10^ However, there are some limitations of fullerene derivatives, such as relatively low lowest unoccupied molecular orbital (LUMO) energy level (≈−3.91 eV^11^) which leads to loss of open circuit voltage (*V*_OC_), weak absorption in the visible region, relatively high cost,^12^ and easy aggregation, which reduces long‐term stability of devices.^13^

Fullerene‐free OSCs (FF‐OSCs)^9^, ^14^, ^15^, ^16^, ^17^, ^18^, ^19^, ^20^ are composed of a p‐type semiconducting polymer or small molecule as an electron donor and an n‐type semiconducting nonfullerene polymer or small molecule as an electron acceptor. Compared with fullerene acceptors, nonfullerene acceptors present some advantages, such as broad and strong absorption, adjustable LUMO energy levels, and long‐term stability. In recent years, FF‐OSCs developed quickly^21^, ^22^, ^23^, ^24^, ^25^, ^26^, ^27^, ^28^, ^29^, ^30^, ^31^, ^32^, ^33^, ^34^, ^35^, ^36^, ^37^, ^38^, ^39^, ^40^, ^41^, ^42^ and achieved PCEs of up to 6.8%.^25^

Although FF‐OSCs potentially have a bright future, compared with fullerene based OSCs, they are still far away from industrial manufacture. Fullerene‐based OSCs can be fabricated on large area (more than 1 cm^2^),^43^, ^44^ with flexible substrates,^45^, ^46^ without expensive transparent electrodes such as indium tin oxide (ITO),^47^, ^48^ without vacuum evaporated electrodes,^49^, ^50^ and under ambient conditions without nitrogen protection.^51^, ^52^ However, almost all FF‐OSCs reported were small area (less than 0.15 cm^2^) on ITO/glass substrates, with vacuum evaporated electrodes and nitrogen protection.^21^, ^22^, ^23^, ^24^, ^25^, ^26^, ^27^, ^28^, ^29^, ^30^, ^35^, ^36^, ^37^, ^38^ Roll‐to‐roll (R2R) fabrication^53^, ^54^, ^55^ is a promising way to fabricate OSCs. Due to fast speed to produce large area, flexible and stable OSCs with ITO‐free, vacuum‐free, and ambient conditions, R2R may have potential for industrial manufacture of FF‐OSCs. However, until now fabrication of FF‐OSCs by the roll‐coating process has been studied little.^56^, ^57^, ^58^

In this work, we report FF‐OSCs based on a small molecule nonfullerene acceptor fabricated by the roll‐coating process. The structure of inverted FF‐OSCs is shown in **Figure** **1**a. The active layer consisted of a polymer donor PBDTTT‐C‐T^59^ and a nonfullerene acceptor DC‐IDT2T (Figure 1b).^30^ After optimization of morphology of the active layer in roll‐coated devices by using different processing solvents and thermal annealing, the best PCE of 1.019% was achieved with a large device area (1 cm^2^), flexible substrates, ITO‐free, vacuum‐free, and preparation under ambient conditions. Moreover, relative to PC_71_BM‐based control devices, the FF‐OSCs exhibited much better stability.

**Figure 1:**
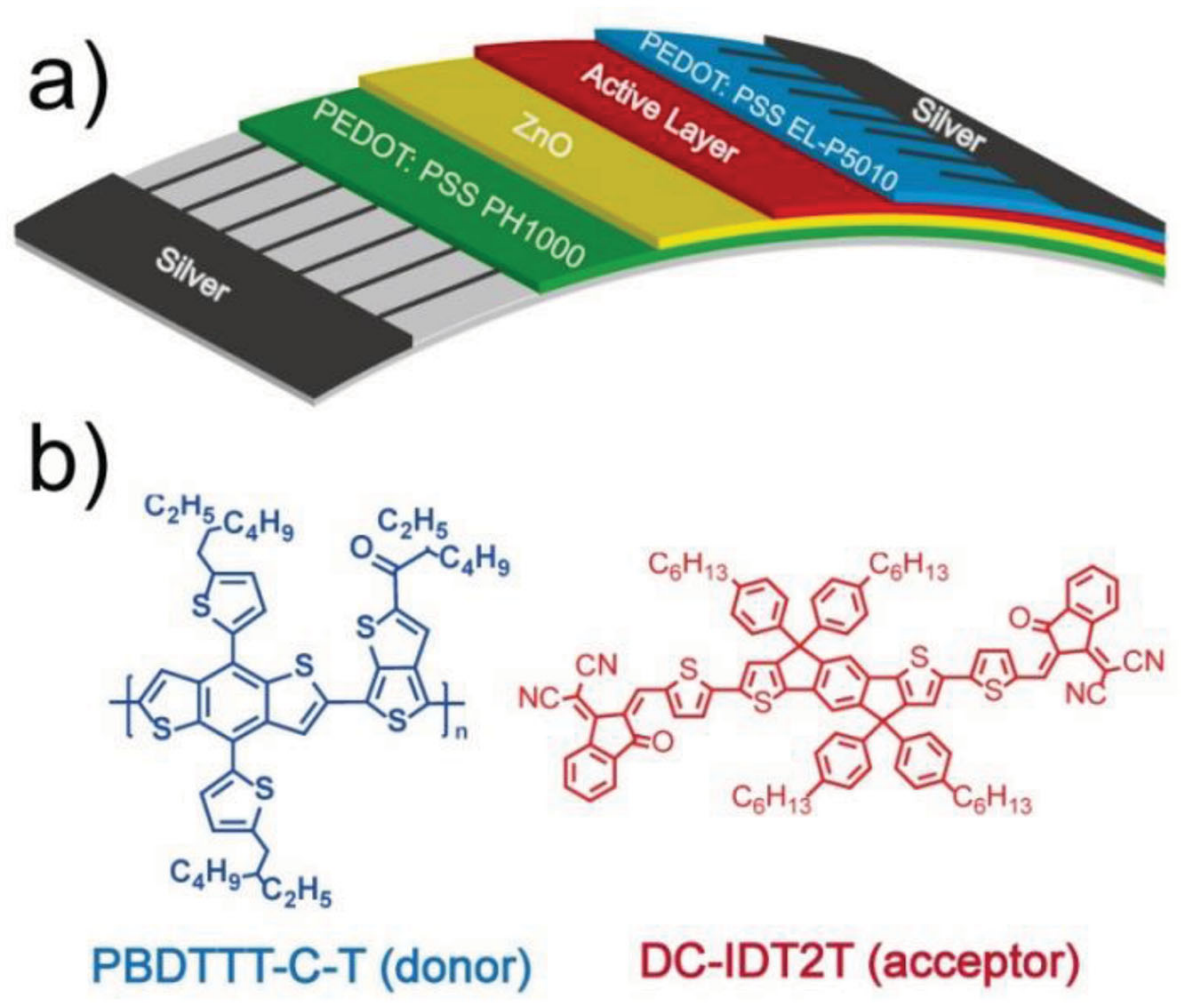
a) Structure of roll‐coated solar cells, and b) molecular structures of PBDTTT‐C‐T (donor) and DC‐IDT2T (acceptor).

These FF‐OSCs were produced by flexographic printing and slot‐die coating on a lab‐scale mini roll‐coater.^60^ The silver (Ag) finger grids (bottom and top layers) were printed using flexo­graphic printing by a patterned rubber roller whereas all other layers were fabricated by slot‐die coating.^50^ As shown in **Figure** **2**a, the flexible substrate consisted of poly(ethyle­neterephthalate) (PET) support layer,^61^ Ag finger grids, conductive layer (poly(3,4‐ethylenedioxythiophene):poly­(styrene­sulfonate), PEDOT:PSS PH1000), and electron transport layer zinc oxide (ZnO). The ink containing polymer donor and nonfullerene acceptor materials was transferred from an external container via a pump to the slot‐die head. The coating width was defined by the width of the head's bottom slot, through which the ink flowed onto the moving substrate. The coating thickness was directed by the ink flow rate and moving speed of the substrate.^52^ We prepared active‐material inks, comprising a blend of the polymer donor PBDTTT‐C‐T and the small mole­cule electron acceptor DC‐IDT2T, using different processing solvents and different donor/acceptor (D/A) ratios, and all solutions had a same concentration of 27 mg mL^−1^ in total. The ink was roll‐coated on four stripes on a flexible PET substrate, precoated with ZnO on high conductive PEDOT:PSS PH1000 cathodic stripes (Figure 2b). The coating was conducted at 60 °C with a web speed of 0.65 m min^−1^, affording a wet thickness in the range of 7–14 μm. The back PEDOT:PSS EL‐P5010 layer was slot‐die coated on the active layer with a further offset of 1 mm (to prevent shorting of the device) (Figure 2c). The coating was conducted at 60 °C with a web speed of 0.8 m min^−1^, affording a wet thickness in the range of 150 μm. The ink formulation and coating took place in ambient air, with the roller kept at 60 °C to facilitate drying of the films for more than 45 min. The Ag electrodes were applied by flexographic printing of a heat curing Ag paste. The Ag paste was added to the flexographic roll and further transferred to the substrate with a web speed of 0.8 m min^−1^ and roll temperature of 60 °C (Figure 2d). Finally, thermal annealing was used for devices at 120 °C for 1 or 2 min. The completed solar cells (Figure 2e) were then divided into ≈320 individual cells each with an active area of 0.9–1.1 cm^2^.

**Figure 2:**
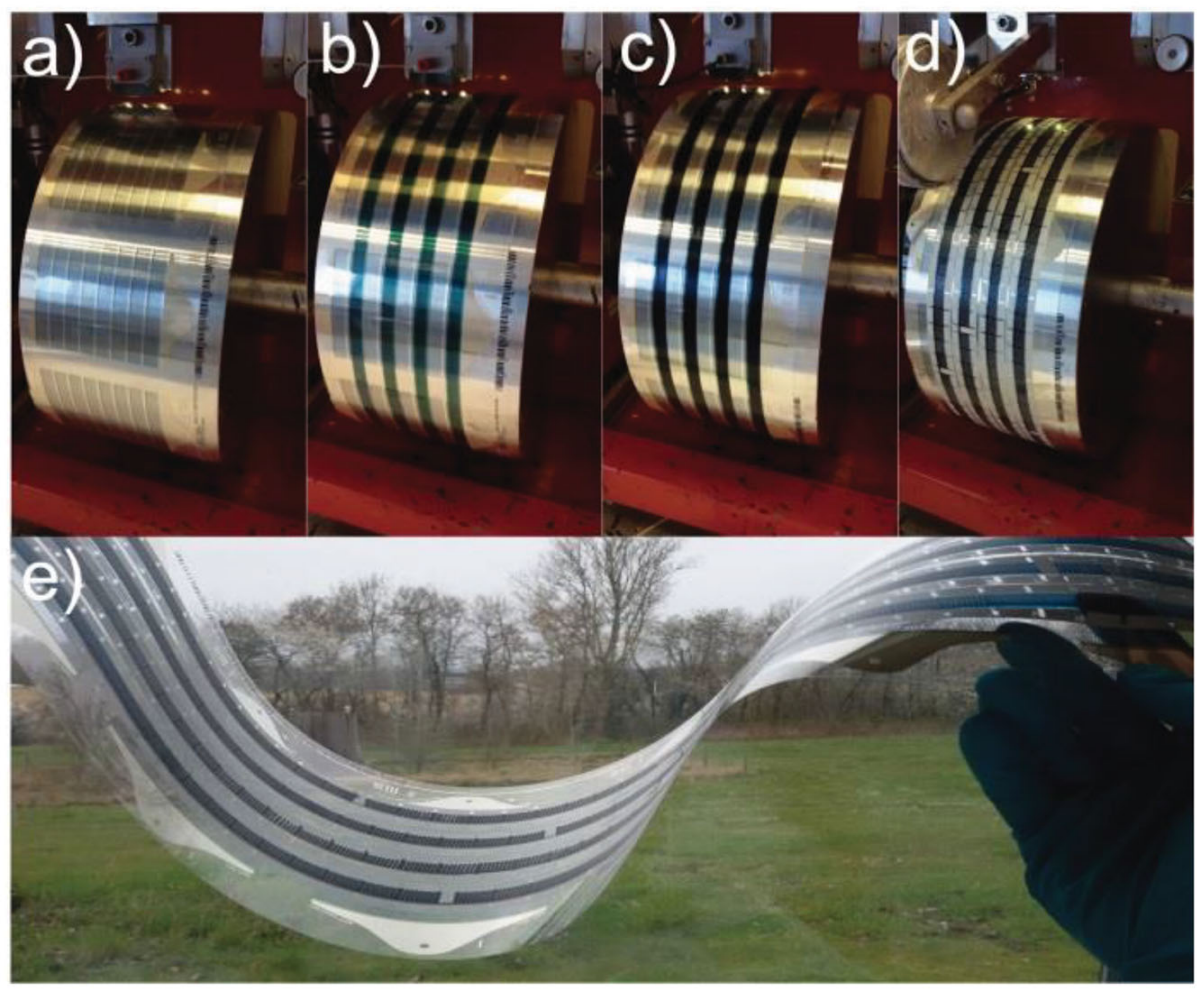
a) PEDOT:PSS PH1000 and ZnO coated PET flexible substrate with Ag finger grid (cathode), b) slot‐die coating of the active layer on the mini roll‐coater, c) slot‐die coating of the PEDOT:PSS EL‐P5010 on the active layer, d) flexographic printing of the Ag electrodes (anode), e) roll‐coated devices fabricated by slot‐die coating.

FF‐OSCs with an inverted structure were fabricated by varying processing solvent, D/A weight ratio, wet thickness of active layer and thermal annealing time. The *J*–*V* curves of FF‐OSCs with different D/A weight ratio, wet thickness of active layer, and thermal annealing time are shown in Figures S1, S2, and S3 of the Supporting Information, respectively. Table S1, Supporting Information, summarizes the average and best device data of FF‐OSCs under different device conditions. The optimized device conditions were D/A weight ratio of 1:1.25, wet thickness of active layer of 9 μm, and thermal annealing at 120 °C for 1 min. **Figure** **3**a,b shows the *J*–*V* and external quantum efficiency (EQE) spectra of FF‐OSCs with different processing solvent (chloroform (CF); *o*‐xylene; chlorobenzene (CB), and dichlorobenzene (DCB)) and thermal annealing under the optimized device conditions. Figure 3c shows the light beam‐induced current (LBIC) image of FF‐OSCs (solvent: CB). LBIC is a well‐established 2D mapping technique for characterization of solar cells and calculation of device area.^62^ This image clearly showed good current response over the whole active region. **Table** **1** shows the average and best device characteristics of FF‐OSCs with different processing solvent and thermal annealing under the optimized device conditions. The processing solvent strongly affected the *V*_OC_, short circuit current density (*J*_SC_) and fill factor (FF). The *V*_OC_ of devices processed with CF, DCB, and CB solvents was similar and much higher than that of *o*‐xylene processed devices. The FF of devices processed with CF and CB was higher than those of *o*‐xylene and DCB processed devices. The devices processed with CB had higher *J*_SC_ and PCE; the average *V*_OC_, *J*_SC_, FF, and PCE of these devices was 0.7748 V, 2.847 mA cm^−2^, 37.22%, and 0.821%, respectively. Thermal annealing led to performance improvement in FF‐OSCs. The solar cells exhibited the best performance after thermal annealing at 120 °C for 1 min; average *V*_OC_ increased from 0.7748 to 0.7983 V, average *J*_SC_ increased from 2.847 to 3.086 mA cm^−2^, average FF increased from 37.22% to 40.11%, and average PCE increased from 0.821% to 0.988%. The best PCE of these devices was 1.019%. As shown in Figure 3b, the trend of EQE was similar to that of *J*_SC_. To evaluate the accuracy of the photovoltaic results, the *J*_SC_ values were calculated from integration of the EQE spectra with the AM 1.5G reference spectrum. The calculated *J*_SC_ was similar to *J*–*V* measurement (the average error was 3.2%, Table 1).

**Table 1:** Average and best device data based on PBDTTT‐C‐T:DC‐IDT2T or PBDTTT‐C‐T:PC_71_BM films with different processing solvent and thermal annealing (the average data were calculated from 10 devices)

Active layer	Processing solvent	*V*_OC_ [V]	*J*_SC_ [mA cm^−2^]	Calculated *J*_SC_ [mA cm^−2^]	FF [%]	PCE [%]	
						Average	Best
PBDTTT‐C‐T:DC‐IDT2T	CF	0.8253	2.089	2.135	37.23	0.642	0.674
PBDTTT‐C‐T:DC‐IDT2T	*o*‐xylene	0.5075	0.502	0.525	27.44	0.070	0.075
PBDTTT‐C‐T:DC‐IDT2T	DCB	0.7836	1.598	1.624	31.78	0.398	0.427
PBDTTT‐C‐T: DC‐IDT2T	CB	0.7748	2.847	2.958	37.22	0.821	0.867
PBDTTT‐C‐T: DC‐IDT2T	CBa)	0.7983	3.086	3.204	40.11	0.988	1.019
PBDTTT‐C‐T: PC_71_BM	DCBb)	0.6292	7.026	7.118	43.59	1.927	2.088

^a)^Annealing at 120 °C for 1 min;

^b)^5% (v/v) 1,8‐diiodooctane (DIO).

**Figure 3:**
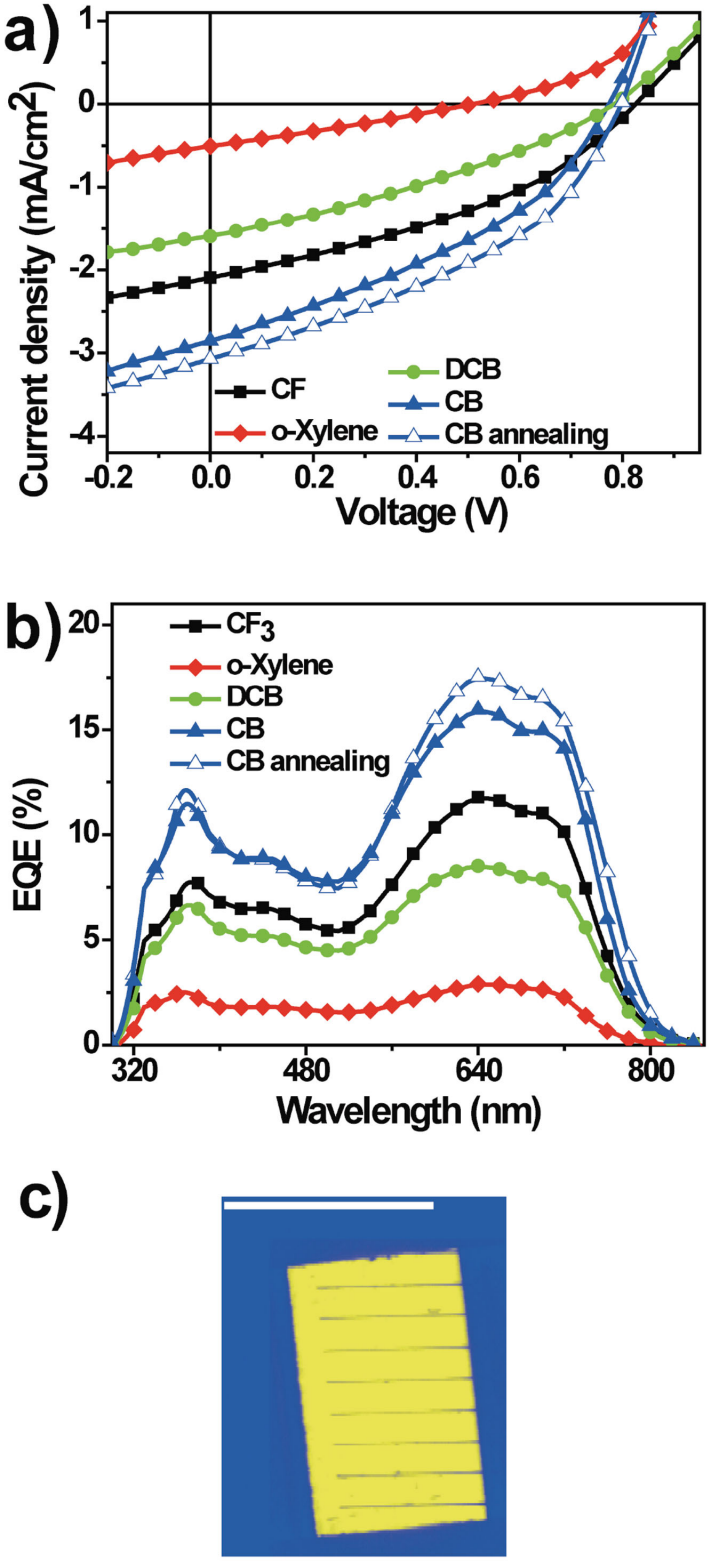
a) *J*–*V* curves and b) EQE spectra of devices with the structure PET/Ag/PEDOT:PSS PH1000/ZnO/PBDTTT‐C‐T:DC‐IDT2T/PEDOT:PSS EL‐P5010/Ag with different processing solvent and thermal annealing under the illumination of an AM 1.5G solar simulator, 100 mW cm^−2^; c) Device area as shown in the LBIC^62^ image, the scale bar is 1 cm.

**Figure** **4** shows the atomic force microscope (AFM) height and phase images of PBDTTT‐C‐T:DC‐IDT2T blend films with different processing solvent and thermal annealing. The blend films with different processing solvent exhibited a typical cluster structure with many aggregated domains and a root‐mean‐square (RMS) roughness of 3.21 nm (DCB, Figure 4a), 2.29 nm (CF, Figure 4c), and 1.64 nm (CB, Figure 4e), respectively. The films became smoother and the RMS roughness decreased from 1.64 to 1.23 nm after thermal annealing (Figure 4g). The more uniform and smoother morphology of PBDTTT‐C‐T:DC‐IDT2T blended films with suitable phase separation was observed with the use of CB and thermal annealing, which was responsible for improved *J*_SC_ and PCE. The blended films processed by *o*‐xylene were not uniform with big aggregation (Figure S4, Supporting Information).

**Figure 4:**
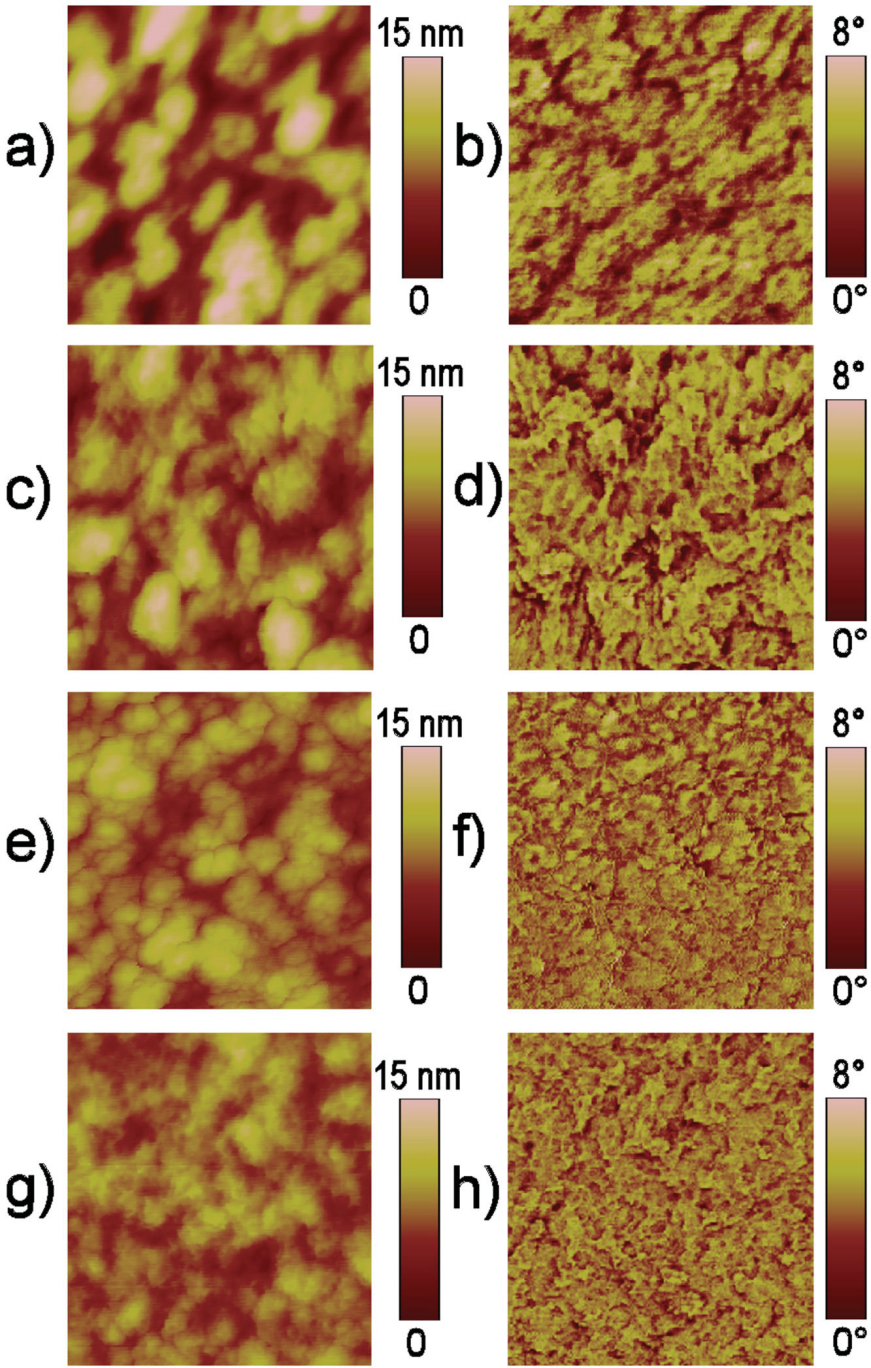
a,c,e,g) AFM height images and b,d,f,h) phase images of PBDTTT‐C‐T:DC‐IDT2T films roll‐coated on PET/Ag/PEDOT:PSS PH1000/ZnO substrates: a,b) DCB; c,d) CF; e,f) CB; g,h) CB with thermal annealing. The scan size of all height images and all phase images is 500 × 500 nm.

The three main qualities that a solar cell must possess are high efficiency, easy fabrication, and good stability. As shown in **Figure** **5**, we measured the stability of PBDTTT‐C‐T/DC‐IDT2T (processing by CB solvent and annealing at 120 °C for 1 min) and PBDTTT‐C‐T/PC_71_BM (processing by DCB solvent and 5% DIO) devices in air under continuous AM1.5G illumination for 180 h. The PCE of PC_71_BM‐based device decreased more quickly than that of nonfullerene acceptor‐based device. The polymer/nonfullerene device showed much better stability (maintained 85% PCE after 180 h) relative to polymer/fullerene device (maintained 50% PCE after 180 h). **Figure** **6** shows the optical microscope images of PBDTTT‐C‐T/DC‐IDT2T films and PBDTTT‐C‐T/PC_71_BM films before and after 180 h stability test. The appearance of PBDTTT‐C‐T/DC‐IDT2T film did not change, while some black dots (≈10 μm) were clearly observed in PBDTTT‐C‐T/PC_71_BM film after 180 h stability test, which decreased the PCE. **Figure** **7** shows the LBIC images of PBDTTT‐C‐T/DC‐IDT2T films and PBDTTT‐C‐T/PC_71_BM films before and after 180 h stability test. The measured current values for each point can be translated into a scale of different color hues and represented as a map showing areas of high and low current response to the incident light.^62^ After 180 h stability test, the LBIC images of PBDTTT‐C‐T/DC‐IDT2T films changed a little, which means little loss of current during the stability test (Figure 7a,b). On the contrary, the color of LBIC images of PBDTTT‐C‐T/PC_71_BM films after the 180 h stability test became darker (Figure 7c,d), which means significant loss of current.

**Figure 5:**
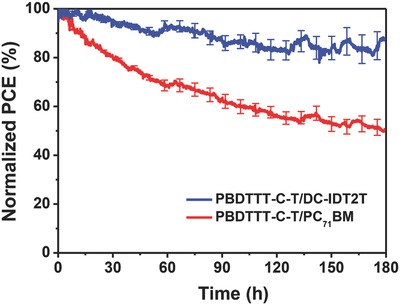
Stability curves of PBDTTT‐C‐T/DC‐IDT2T and PBDTTT‐C‐T/PC_71_BM devices under continuous AM 1.5G illumination for 180 h.

**Figure 6:**
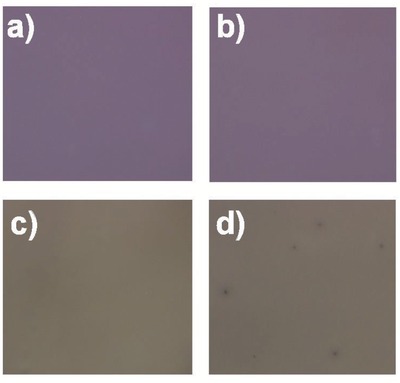
Optical microscope images of a,b) PBDTTT‐C‐T/DC‐IDT2T and c,d) PBDTTT‐C‐T/PC_71_BM films a,c) before and d,b) after exposing to sunlight for 180 h. The scan size of all images is 500 × 500 μm.

**Figure 7:**
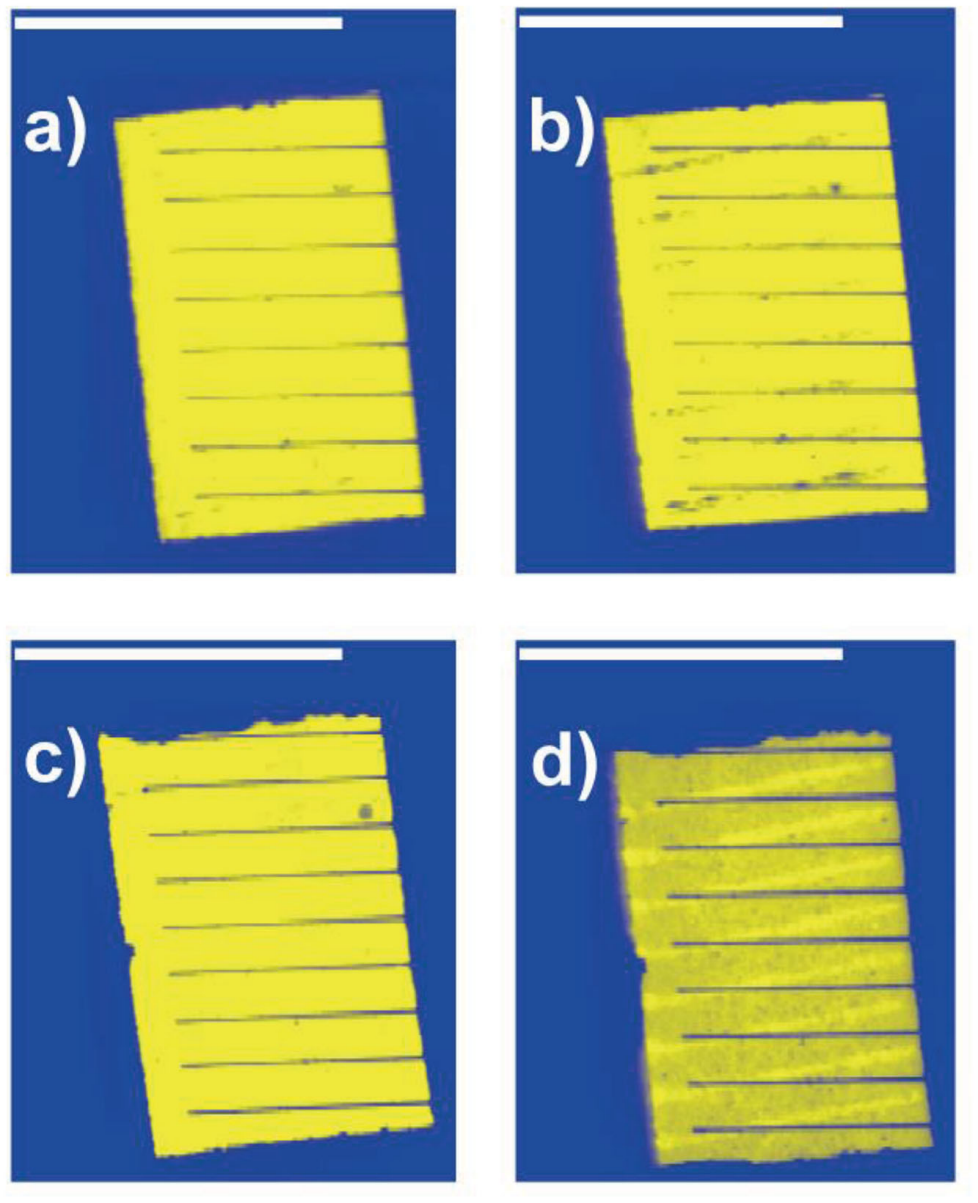
LBIC images of a,b) PBDTTT‐C‐T/DC‐IDT2T and c,d) PBDTTT‐C‐T/PC_71_BM films a,c) before and b,d) after exposing to sunlight for 180 h. All the scale bars are 1 cm.

In summary, we report FF‐OSCs based on polymer donor PBDTTT‐C‐T and nonfullerene small molecule acceptor DC‐IDT2T fabricated by the roll‐coating process. The solar cells fabricated using processing solvent CB and thermal annealing exhibited the best performance due to the uniform and smooth surface of the blended film. The best PCE of 1.019% was achieved with large device area (1 cm^2^), flexible substrates, ITO‐free, vacuum‐free, and ambient conditions. The nonfullerene‐based device showed much better stability than the fullerene‐based control device. Our preliminary results demonstrate that nonfullerene small molecule acceptors could be promising for industrial manufacture of high‐performance OSCs.

## Supporting information

As a service to our authors and readers, this journal provides supporting information supplied by the authors. Such materials are peer reviewed and may be re‐organized for online delivery, but are not copy‐edited or typeset. Technical support issues arising from supporting information (other than missing files) should be addressed to the authors.

Supplementary
