# Supplementary material for: Roll‐Coated Fabrication of Fullerene‐Free Organic Solar Cells with Improved Stability
Source: Adv Sci (Weinh). 2015 Apr 28;2(6):1500096. doi: 10.1002/advs.201500096 (PMC5115398; doi:10.1002/advs.201500096)
Supplement: Supplementary file 1 — Supplementary [file ADVS-2-0k-s001.pdf]

Copyright WILEY-VCH Verlag GmbH & Co. KGaA, 69469 Weinheim, Germany, 2015.

ADVANCED SCIENCE
Open Access

Supporting Information

for *Adv. Sci.*, DOI: 10.1002/advs.201500096

Roll-Coated Fabrication of Fullerene-Free Organic Solar Cells with Improved Stability

Pei Cheng, Huitao Bai, Natalia K. Zawacka, Thomas R. Andersen, Wenqing Liu, Eva Bundgaard, Mikkel Jørgensen, Hongzheng Chen, Frederik C. Krebs, and Xiaowei Zhan**

Supporting Information for

Roll-Coated Fabrication of Fullerene-Free Organic Solar Cells with Improved Stability

By Pei Cheng, Huitao Bai, Natalia K. Zawacka, Thomas R. Andersen, Wenqing Liu, Eva Bundgaard, Mikkel Jørgensen, Hongzheng Chen, Frederik C. Krebs* and Xiaowei Zhan*

1. Experimental details

1.1 Materials

Unless stated otherwise, solvents and chemicals were obtained commercially and were used without further purification. PBDTTT-C-T[1] was purchased from Solamer Materials Inc, and DC-IDT2T[2] was synthesized according to our previous report. Chlorobenzene (CB), dichlorobenzene (DCB), chloroform (CF) and o-xylene were obtained from J&K Chemical Inc. The polyethylene terephthalate (PET) substrate was Melinex ST506 obtained from Dupont–Teijin.[3] The PET/silver (Ag) substrates were prepared according to the literature procedures.[4] The metal grid had a finger pattern. Two types of highly conductive poly(3,4-ethylenedioxythiophene): poly(styrenesulfonate) (PEDOT: PSS) were employed. Clevios PH1000 from Heraeus was used on the bottom Ag diluted with isopropyl alcohol (IPA) in the ratio 10:3 (w/w). Orgacon EL-P5010 was used as top PEDOT: PSS diluted with IPA in a 2:1 (w/w) ratio. Zinc oxide (ZnO) nanoparticles in acetone with a concentration of 55 mg ml^{-1} were employed for the electron transport layer. The silver electrodes were printed using a flexographic printing silver paste (bottom: PFI-722 from PChem Associates, top: 5025 from Dupont). The front PEDOT:PSS electrode had a sheet resistivity of 60–90 $\Omega\ \square^{-1}$, and the substrate with metal grid had a sheet resistivity of 10 $\Omega\ \square^{-1}$. The sheet resistivity of the back PEDOT:PSS electrode was 60 $\Omega\ \square^{-1}$ on its own and the sheet resistivity of the top silver electrode was 0.1 $\Omega\ \square^{-1}$. The PET substrate with Ag grid, PEDOT:PSS layer and ZnO layer

had the maximum transmittance of 74.8% at 436 nm, and the transmittance at 550 nm was 70.7%.

1.2 Characterization of flexible solar cells.

Solar cells were measured with a Keithley 2400 source meter under a KHS 575 solar simulator with an AM 1.5G 100 mW cm^{-2} intensity. The EQE spectrum was measured using a Solar Cell Spectral Response Measurement System QEX10. The Light beam induced current (LBIC) experiments were carried out using a custom made setup with 405 nm laser diode (5 mW output power, 100 μm spot size ≈65 W cm^{-2}, ThorLabs)[5] mounted on a computer controlled XY-stage and focused to a spot size of <100 μm. The short circuit current from the device under study was measured using a computer controlled source measure unit (SMU, a Keithley 2400 instrument). A custom written computer program was used to scan the solar cell devices in a raster pattern in 200 μm steps in the X and the Y directions, logging the coordinates and measured current. The results were then converted to yellow/blue colored bitmaps in 255 different hues with another custom written program. Bright yellow represents the highest absolute current extracted while blue represents the lowest current. Current profiles along selected directions were taken from these maps to visualize the relative differences in different regions. The nanoscale morphology of blend film was observed using a Veeco Nanoscopy V atomic force microscopy (AFM) in tapping mode. Optical microscopy images were observed using an Olympus BX51 optical microscope. Stability test of solar cells was carried out under continuous illumination of a KHS 575 solar simulator with an AM 1.5G 100 mW cm^{-2} intensity, and the *J-V* was measured using a Keithley 2400 source meter every 5 min in 180 h. The devices for stability test, optical microscope and LBIC measurements were sealed by cover films (2 glass slides with a UV curable epoxy resin) and exposed under the solar simulator (AM1.5G, 1000 W m^{-2}, 75 ± 5° C, 40 ± 10% relative humidity) for 180 h. These tests were carried out in winter and inside room.

2. Details of device optimization

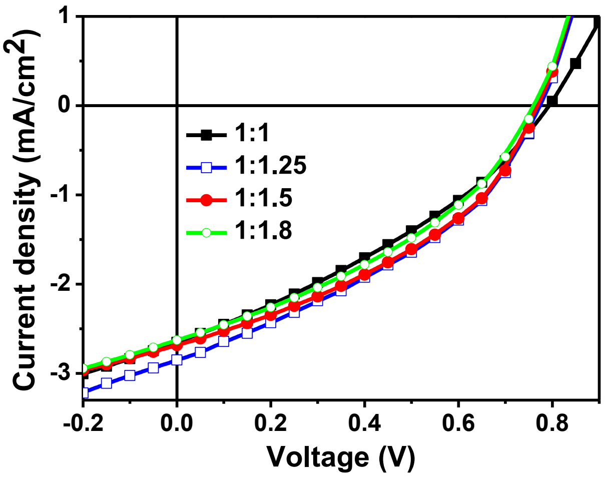

Figure S1. *J–V* curves of devices with the structure PET/Ag/PEDOT: PSS PH1000/ZnO/PBDTTT-C-T: DC-IDT2T/PEDOT: PSS 5010/Ag with different D/A ratio under the illumination of an AM 1.5G solar simulator, 100 mW cm^{-2}.

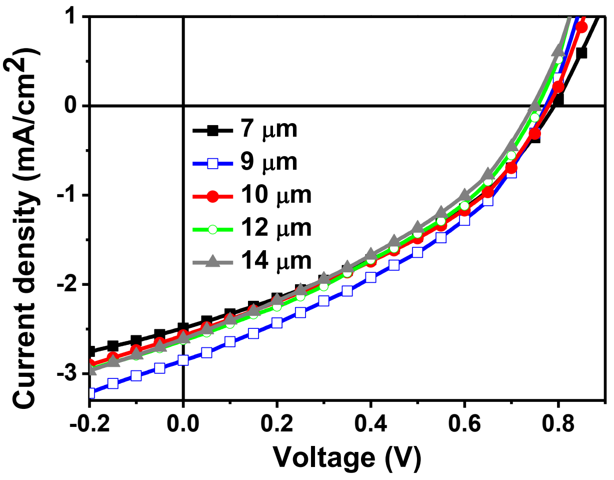

Figure S2. *J–V* curves of devices with the structure PET/Ag/PEDOT: PSS PH1000/ZnO/PBDTTT-C-T: DC-IDT2T/PEDOT: PSS 5010/Ag with different wet thicknesses of active layer under the illumination of an AM 1.5G solar simulator, 100 mW cm^{-2}.

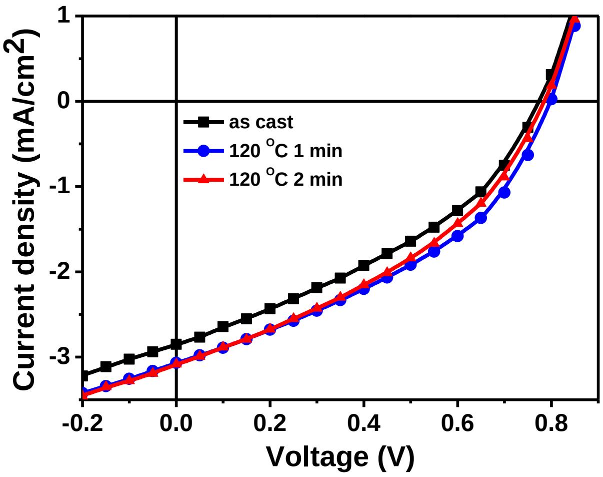

Figure S3. *J–V* curves of devices with the structure PET/Ag/PEDOT: PSS PH1000/ZnO/PBDTTT-C-T: DC-IDT2T/PEDOT: PSS 5010/Ag with different thermal annealing time under the illumination of an AM 1.5G solar simulator, 100 mW cm^{-2}.

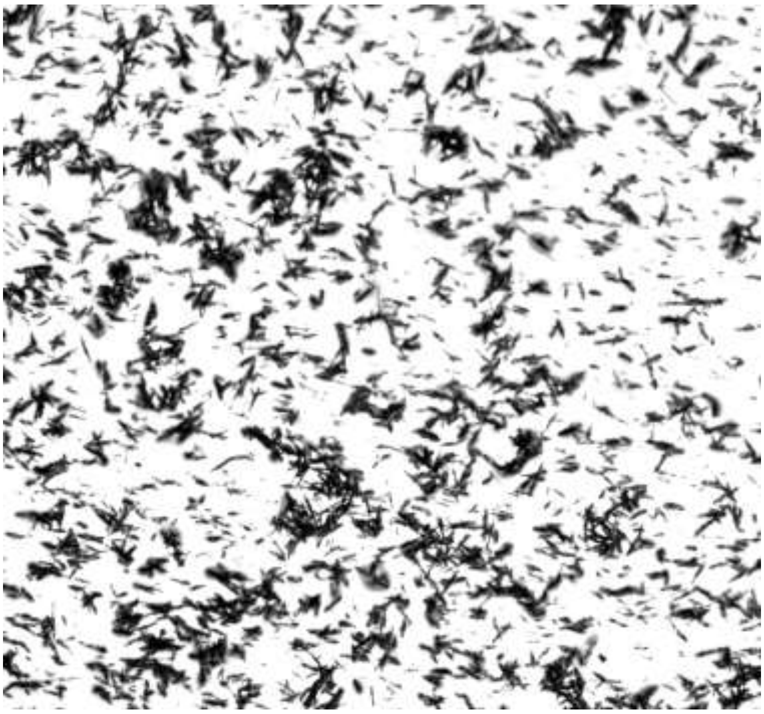

Figure S4. Optical microscope image of PBDTTT-C-T: DC-IDT2T films roll-coated on PET/Ag/PEDOT: PSS PH1000/ZnO substrates with o-xylene solvent. The scan size is 800 μm × 800 μm.

Table S1. Average and best device data based on PBDTTT-C-T: DC-IDT2T films under different device conditions

Solvent	D/A ratio	Wet thickness of active layer (μm)	Thermal annealing	V_{OC} (V)	J_{SC} (mA cm^{-2})	FF (%)	PCE (%)	
							average	best
CF	1:1.25	9		0.8253	2.089	37.23	0.642	0.674
o-xylene	1:1.25	9		0.5075	0.502	27.44	0.070	0.075
DCB	1:1.25	9		0.7836	1.598	31.78	0.398	0.427
CB	1:1	9		0.7933	2.652	33.44	0.704	0.732
CB	1:1.25	7		0.7914	2.475	37.11	0.727	0.758
CB	1:1.25	9		0.7748	2.847	37.22	0.821	0.867
CB	1:1.25	9	120 °C 1 min	0.7982	3.086	40.11	0.988	1.019
CB	1:1.25	9	120 °C 2 min	0.7852	3.102	37.98	0.925	0.965
CB	1:1.25	10		0.7798	2.580	37.02	0.745	0.790
CB	1:1.25	12		0.7602	2.631	36.16	0.723	0.746
CB	1:1.25	14		0.7508	2.612	35.15	0.689	0.701
CB	1:1.5	9		0.7696	2.660	38.93	0.797	0.832
CB	1:1.8	9		0.7628	2.624	37.07	0.742	0.789

Table S2. Average and best device data based on PBDTTT-C-T: $PC_{71}BM$ films under different device conditions

Solvent	DIO (v/v %)	V_{OC} (V)	J_{SC} (mA cm^{-2})	calculated J_{SC} (mA cm^{-2})	FF (%)	PCE (%)	
						average	best
CF	0	0.7018	3.543	3.601	32.01	0.760	0.773
o-xylene	0	0.3657	2.467	2.544	27.88	0.252	0.287
CB	0	0.6631	4.198	4.215	29.51	0.821	0.838
DCB	0	0.6891	5.474	5.512	34.84	1.314	1.369
DCB	3	0.6501	6.629	6.683	41.22	1.776	1.792
DCB	5	0.6292	7.026	7.118	43.59	1.927	2.088
DCB	7	0.6062	6.289	6.311	41.23	1.572	1.625

References:

[1] L. J. Huo, S. Q. Zhang, X. Guo, F. Xu, Y. F. Li, J. H. Hou, *Angew. Chem.-Int. Edit.* **2011**, *50*, 9697.

[2] H. Bai, Y. Wang, P. Cheng, J. Wang, Y. Wu, J. Hou, X. Zhan, *J. Mater. Chem. A* **2015**, *3*, 1910.

[3] M. Hösel, R. R. Søndergaard, M. Jørgensen, F. C. Krebs, *Energy Technology*, **2013**, *1*, 102.

[4] H. F. Dam, F. C. Krebs, *Sol. Energy Mater. Sol. Cells*, **2012**, *97*, 191.

[5] F. C. Krebs, M. Jørgensen, *Adv. Optical Mater.*, **2014**, *2*, 465.
